# Non—leukemic myeloid sarcomas involving only the central nervous system: a case report and literature review

**DOI:** 10.3389/fonc.2026.1755300

**Published:** 2026-03-30

**Authors:** Weixin Han, Hongyan He, Liheng Zheng, Chaoxing Liu, Yanqiang Chen

**Affiliations:** 1 Department of Neurology, Hebei Chest Hospital, Shijiazhuang, Hebei, China; 2 Hebei Provincial Key Laboratory of Pulmonary Disease, Shijiazhuang, Hebei, China; 3 Beijing Chest Hospital, Capital Medical University, Beijing, China; 4 Beijing Tuberculosis and Thoracic Tumor Research Institute, Beijing, China; 5 Shijiazhuang People's Hospital, Shijiazhuang, Hebei, China

**Keywords:** central nervous system, diagnosis, intrathecal injection, leukemia, myeloid sarcoma

## Abstract

A previously healthy 39–year-old male presented with a five-month history of dizziness and progressive numbness and weakness of the lower limbs for more than two months. Contrast-enhanced MRI revealed multiple enhancing soft tissue masses in the spinal canal, while PET/CT indicated numerous hypermetabolic lesions in the intracranial and spinal regions. Initial pathological findings suggested inflammatory lesions, and the diagnosis remained uncertain after consultations at multiple hospitals. The patient was admitted to our hospital on June 15, 2019. Subsequent examinations after admission ruled out tuberculosis infection. A lumbar puncture was performed, and cerebrospinal fluid was collected for cytological examination, revealing numerous abnormal cells. We suspected the presence of a tumor. After intrathecal chemotherapy, the patient’s symptoms showed some improvement; however, subsequent imaging revealed progression of the intracranial lesions accompanied by cerebral herniation. A repeat surgical biopsy suggested myeloid sarcoma (MS). The patient was transferred to a cancer hospital for chemotherapy but succumbed to the critical illness on September 16, 2019. Through this case report, we hope to gain a better understanding of myeloid sarcoma. Timely diagnosis and appropriate treatment are essential for obtaining the desired results.

## 1 Introduction

Myeloid sarcoma (MS) is a rare malignant solid tumor composed of primitive myeloid cells. MS may occur in any organ, most commonly involving soft tissue and the musculoskeletal system, such as the bone, periosteum, lymph nodes, and skin, but MS in the breast, ovary, rectum, pancreas, and bladder has also been reported in various studies (1–5). MS involving the central nervous system is very rare, accounting for only3.25% of MS cases (6, 7). MS is most common in acute myeloid leukemia (AML), myeloproliferative neoplasm (MPN), or myelodysplastic syndrome (MDS) and usually occurs during the course of active leukemia or after remission (8). It can also occur in isolation without any evidence of hematological disease, and its incidence is about two out of 1, 000, 000 (9, 10). Therefore, very few MS cases are not associated with hematological diseases and affect only the central nervous system. They have only been mentioned in individual reports (11, 12).

Here, we report a patient suffering from MS that affected only the central nervous system. The patient ‘s hematological examination was normal during hospitalization. This was the first MS patient in our hospital. We analyzed the patient ‘s clinical manifestations, imaging characteristics, histopathological characteristics, and treatment measures to learn about the disease, gain a better understanding, and obtain experience for future clinical diagnosis and treatment.

## 2 Case report

**Figure 1:**
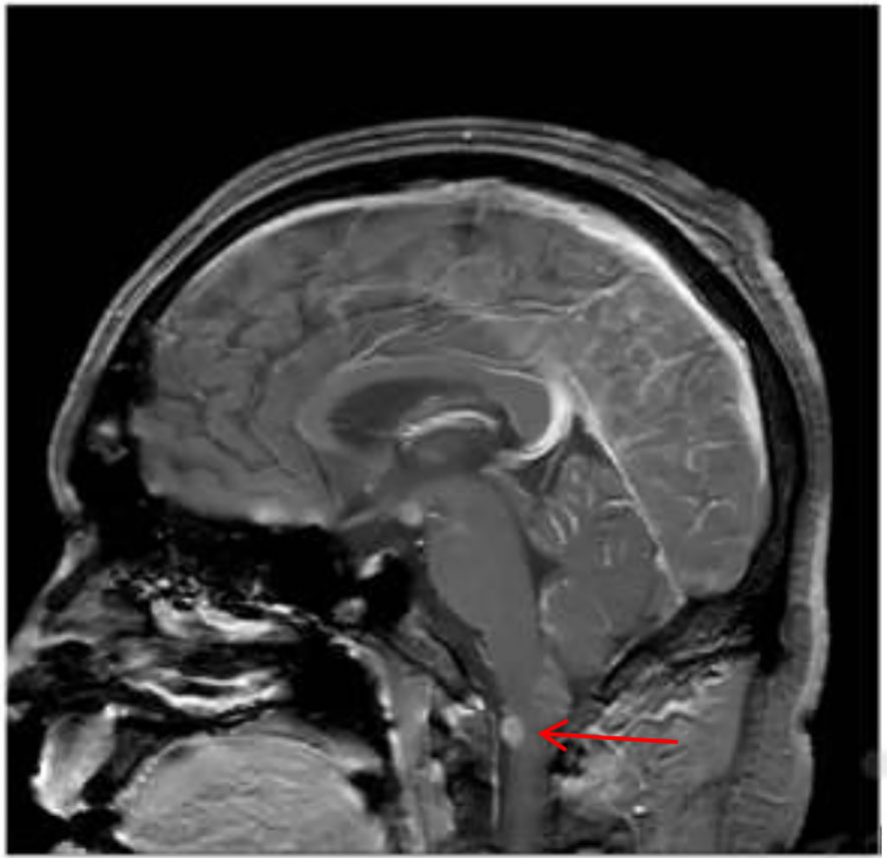
Brain magnetic resonance enhancement image indicated an abnormalenhancement signal in the cervical spinal cord.

**Figure 2:**
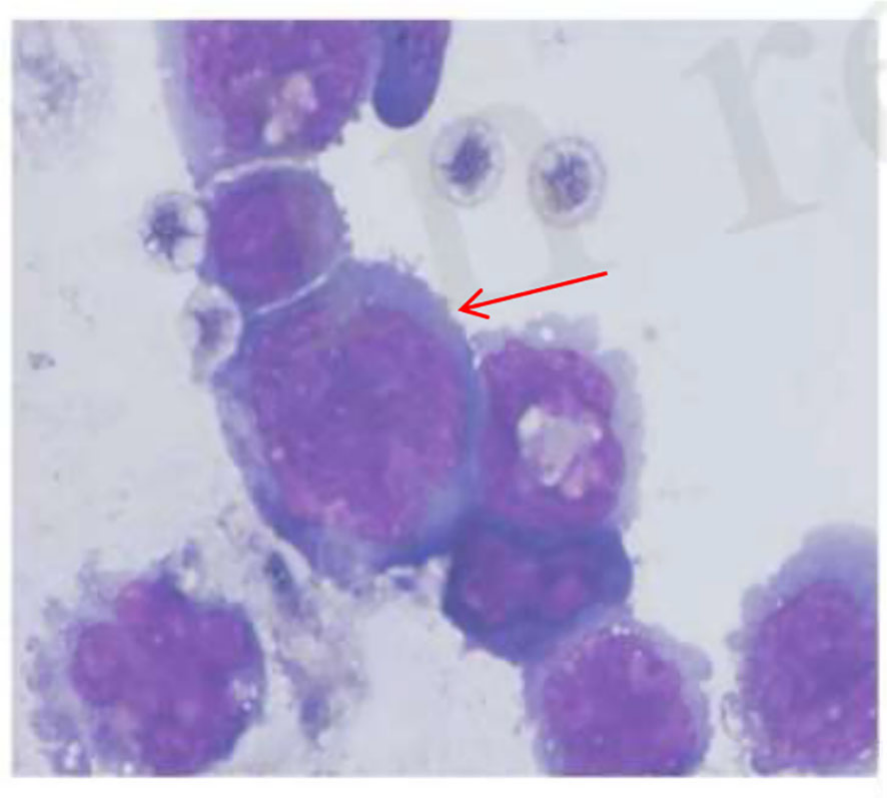
Cerebrospinal fluid cytology: A large number of abnormal cells were visible (MGG×1000).

**Figure 3:**
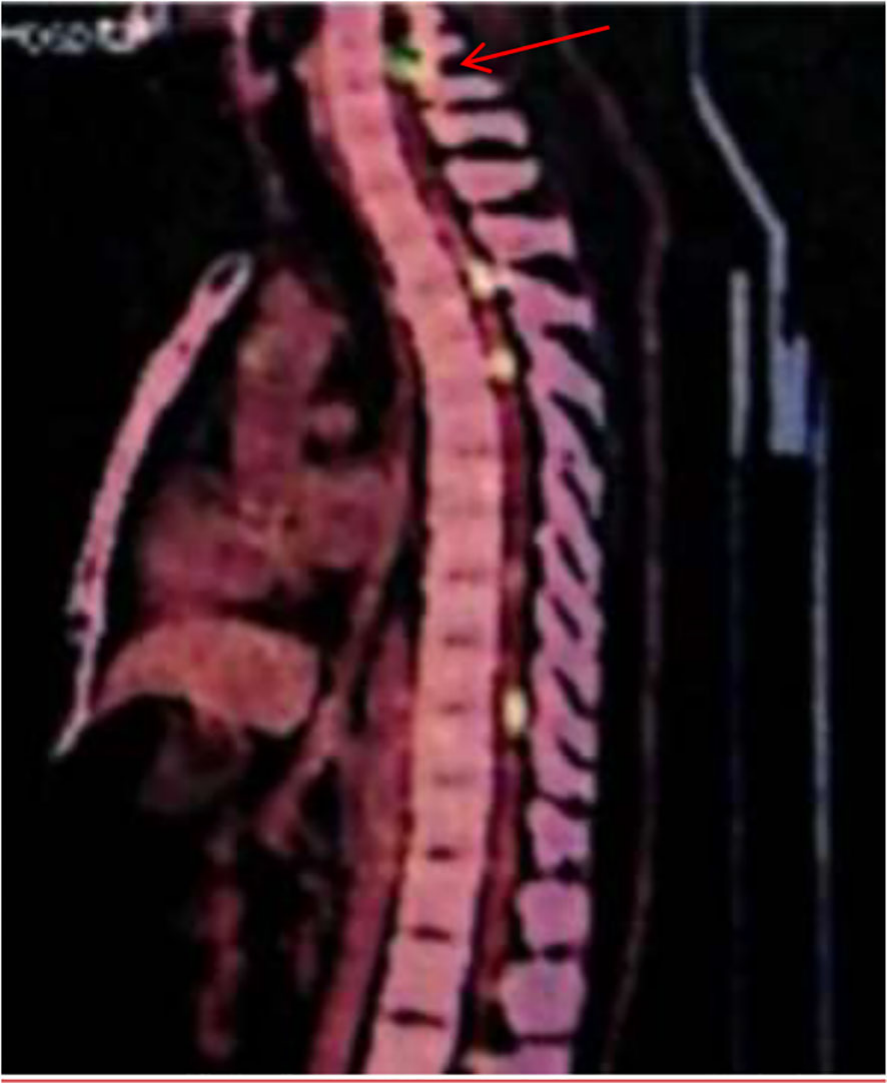
PET/CT: Multiple abnormally high metabolic signals were detected in the spinal cord.

A 39-year-old man went to the hospital for dizziness, headache, tinnitus, and hearing loss in October 2018 and was hospitalized for treatment. No abnormalities were found in cranial transcerebral CT, neck vascular ultrasound, and transcranial Doppler examinations. In January 2019, his symptoms continued to worsen, and he developed chest tightness, dyspnea, progressive limb weakness, and bowel and bladder dysfunction. On May 13, 2019, he underwent functional MRI of his head and entire spinal cord, which showed multiple abnormal uniformly enhanced signal shadows in the brain parenchyma, part of the meninges and spinal cord, suggesting the possibility of neurofibroma (**Figure 1**). During this period, he also underwent lumbar puncture, and the cerebrospinal fluid was collected and sent for cytological examination, which revealed a large number of suspicious atypical cells (**Figure 2**). He further underwent PET/CT examination, and the results showed multiple hypermetabolic lesions in the brain and spinal canal (**Figure 3**), and the possibility of lymphoma was considered. He subsequently underwent spinal cord biopsy, and the histopathological results showed inflammatory lesions, suggesting neurological sarcoidosis. He then received 7 days of high-dose hormone shock therapy.

**Figure 4:**
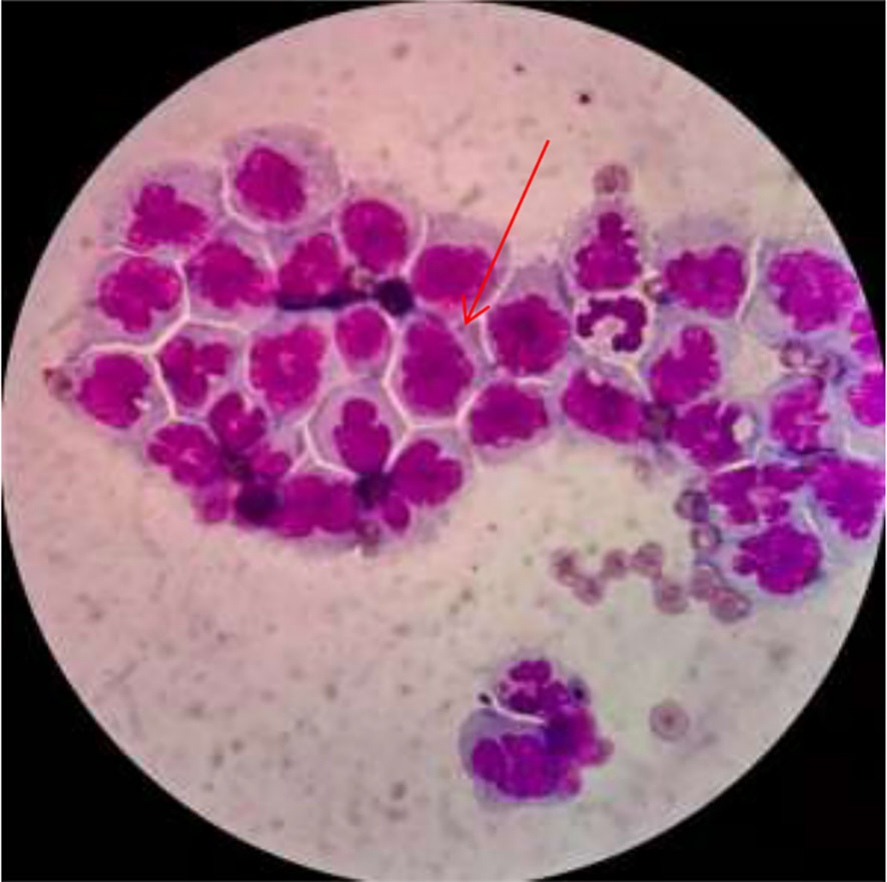
Cerebrospinal fluid cytology: A large number of heterotypic cells were observed. The cell membrane had irregular edges, most of the nuclei were petal-like, nucleoli were visible, and the cytoplasm appeared blue.

**Figure 5:**
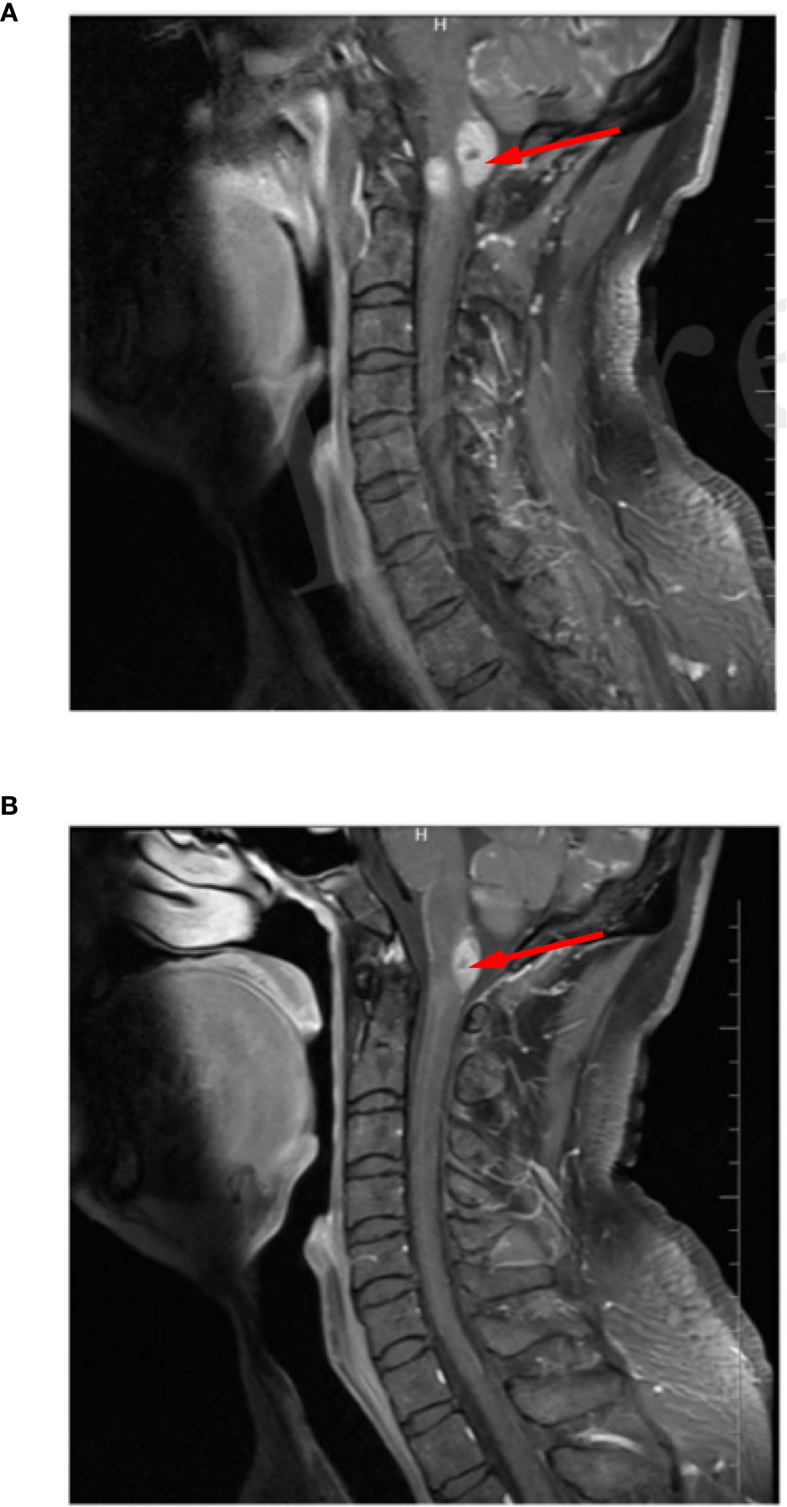
**(A)** Cerebral nuclear magnetic resonance enhancement before intrathecalchemotherapy revealed multiple abnormal enhancement signals in the cervical spinalcord. **(B)** After intrathecal chemotherapy, the abnormal enhancement signal at this sitewas slightly smaller than before.

Because he felt that his clinical symptoms were relieved, he subsequently underwent a re-examination of the brain MRI enhanced scan, but the results showed that the intracranial lesions had progressed significantly compared with before, and the enhancement was diffuse meningeal type. Considering that the possibility of tuberculous meningoencephalitis could not be ruled out, he was transferred to the Department of Neurology of our hospital on June 15, 2019. While hospitalized he underwent another lumbar puncture. The cerebrospinal fluid was collected for testing of tuberculosis infection, including acid-fast staining, tuberculosis DNA testing, XPERT gene testing, and tuberculosis culture. None of these tests detected Mycobacterium tuberculosis, but the cerebrospinal fluid cytology still showed a large number of atypical cells (**Figure 4**). We conducted a routine examination of all his systemic organs again, and no significant abnormal lesions were found. Meanwhile, he underwent bone marrow aspiration, and the bone marrow morphology analysis indicated hypercellularity with active proliferation of nucleated cells. All stages of the granulocytic series were observed, predominantly myelocytes and metamyelocytes, with occasional giant metamyelocytes. The erythroid series was mainly composed of intermediate and late-stage normoblasts, and binucleated early erythroblasts were visible. Mature red blood cells showed no significant changes in size or morphology. Megakaryocytes numbered 119, and platelet clusters were readily observed. His cerebrospinal fluid was sent for flow cytometric analysis, which revealed a small number of lymphocytes among the nucleated cells in the cerebrospinal fluid, with a decreased CD4/CD8 ratio (0.64) in T cells. B cells showed missing surface K/L chain expression. Monocytes were frequently seen, with absent surface HLA-DR expression, and the proportion of granulocytes was increased. Based on all the aforementioned findings, we believe he is suffering from a rare tumor originating from the central nervous system. We conducted thorough discussions with the patient and his family regarding the disease condition. Both the patient and his family were unable to accept systemic chemotherapy but agreed to trial intrathecal chemotherapy (consisting of intrathecal injections of 10 mg methotrexate three times per week). Following this treatment, his clinical symptoms gradually improved. A follow-up contrast-enhanced cranial MRI also showed a marked reduction in the size of the enhancing nodular lesions within the spinal cord (**Figures 5a, b**).

**Figure 6:**
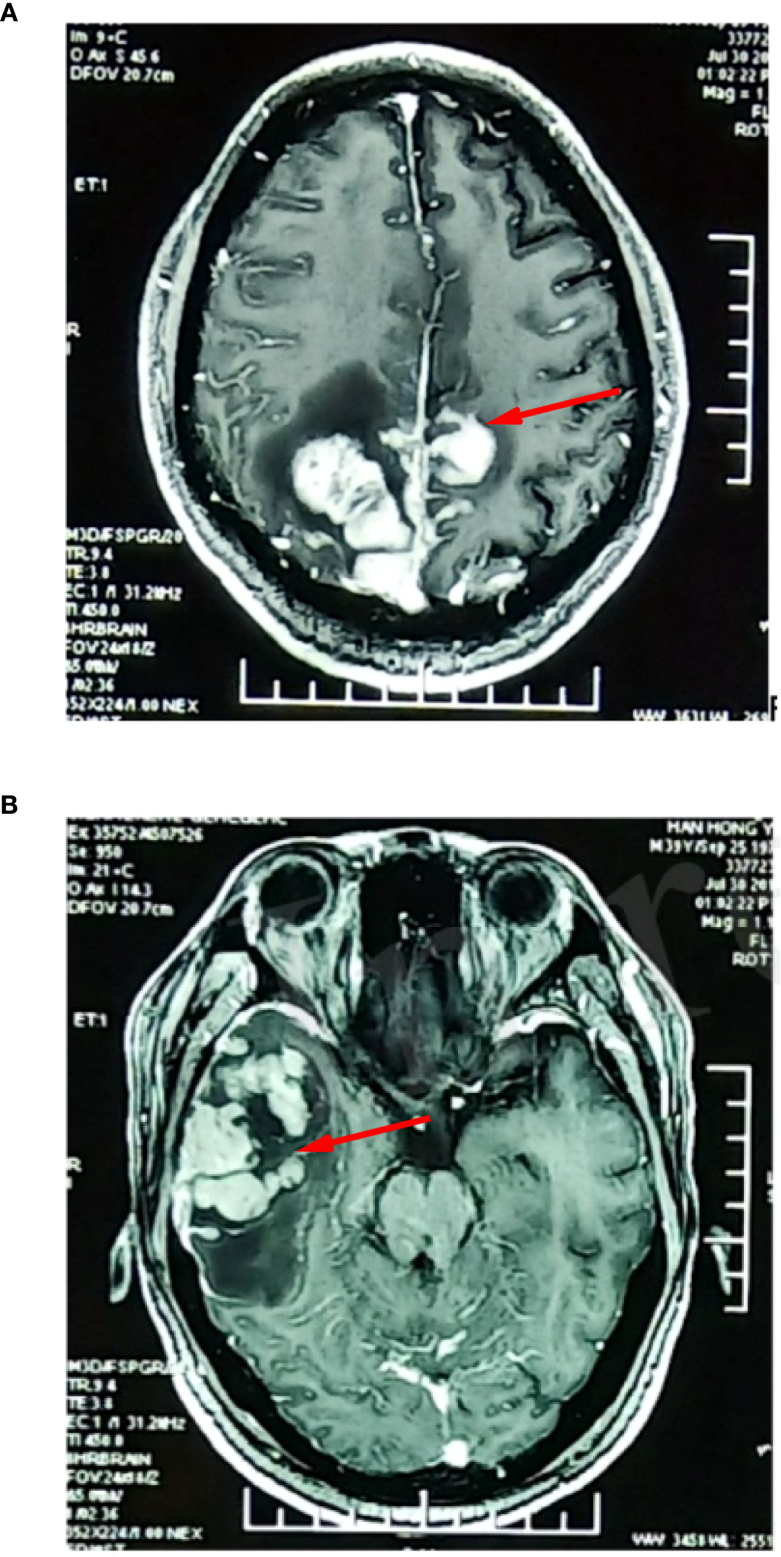
Re-examination of craniocerebral nuclear magnetic resonance enhancement indicated a marked increase in intracranial lesions. **(A)** represents the lesion in the occipital lobe, while **(B)** represents the lesion in the temporal lobe.

**Figure 7:**
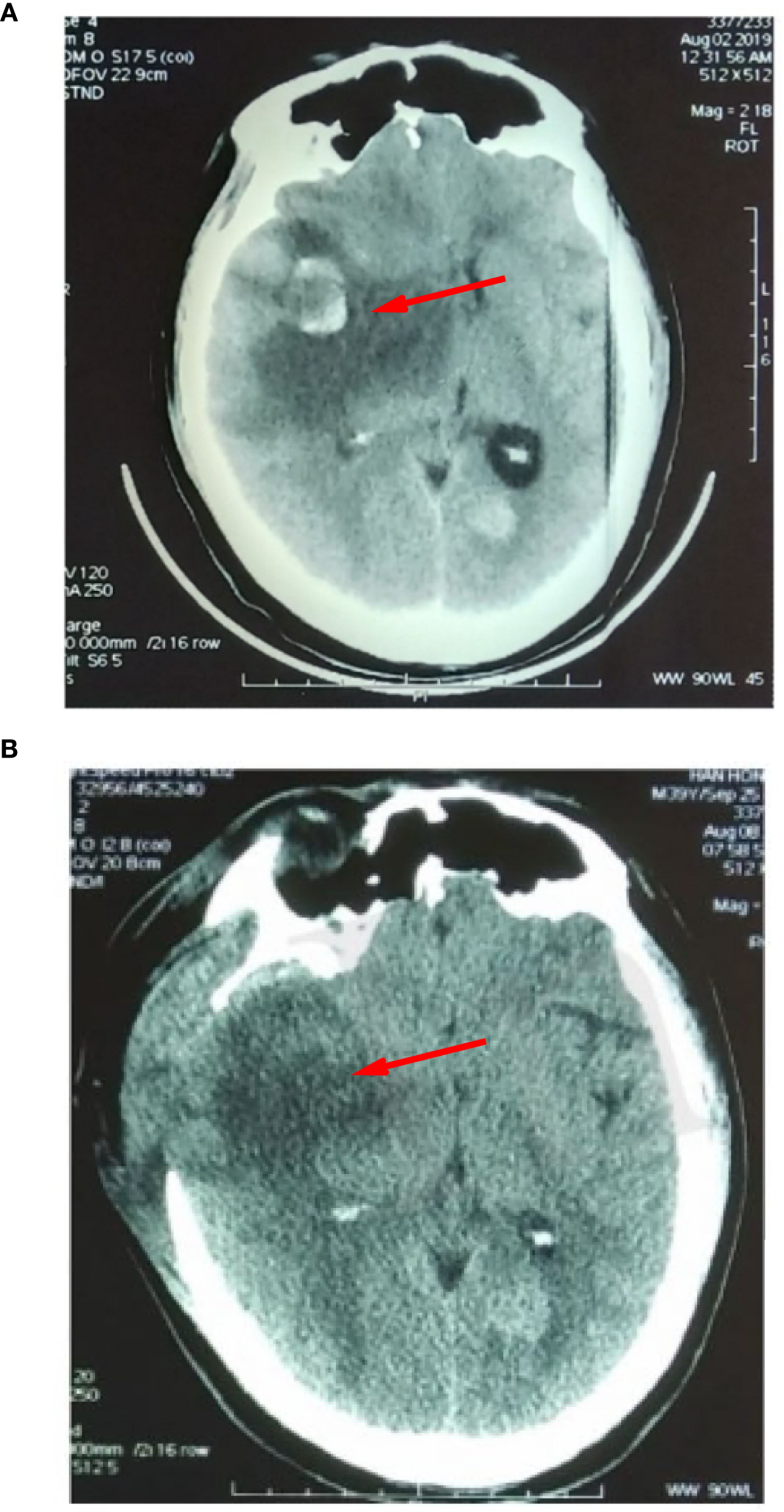
**(A)** Intracranial lesion spreading and cerebral hernia were detected. **(B)** Imagetaken after decompression of the skull flap.

**Figure 8:**
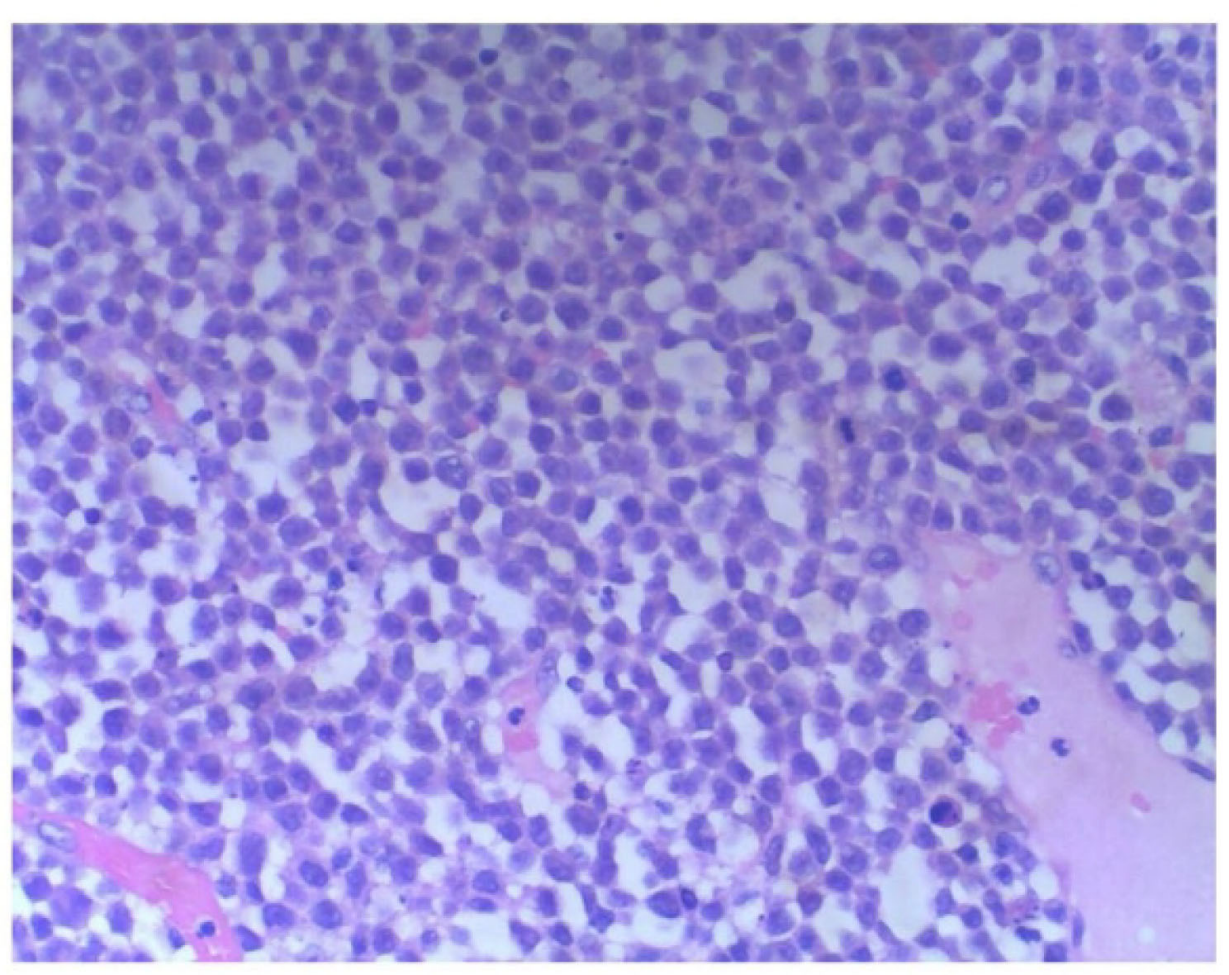
Histopathology shows: diffuse proliferation of primitive cells, relatively consistent cell size, high nuclear-cytoplasmic ratio, visible small nucleoli, easily visible mitotic figures, and nuclear debris.

On July 20, 2019, he continued treatment at another hospital, during which intrathecal chemotherapy was not resumed. A follow-up contrast-enhanced cranial MRI revealed a significant enlargement of the intracranial lesions and increased meningeal enhancement compared to previous findings (**Figure 6**). He subsequently developed cerebral herniation and urgently underwent decompressive craniectomy and brain tissue biopsy (**Figure 7**). Immunohistochemical analysis of the biopsy tissue showed positivity for Bcl-2(+), scattered positivity for Bcl-6(+), focal positivity for CD10(+), negativity for CD17 (–), CD163(-), CD20(-), CD3(-), scattered positivity for CD30(+), negativity for CD34(-), positivity for CD68(+), negativity for CD7(-), positivity for CD99(+), negativity for CKpan(-), a Ki-67 index of 95%, negativity for LCA(-), negativity for MUM-1(-), negativity for PAX5(-), negativity for TdT(-), and scattered positivity for Vimentin(+). Based on the pathological morphology and immunophenotype, the findings were consistent with a malignant tumor of lymphohematopoietic origin, suggestive of myeloid sarcoma (**Figure 8**). Unfortunately, the patient passed away on September 16, 2019, after receiving only one cycle of high-dose cytarabine chemotherapy.

## 3 Discussion

Myeloid sarcoma (MS) is a type of extramedullary leukemia formed by the proliferation and infiltration of primitive or immature myeloid cells outside the bone marrow (13, 14). MS can occur at any age, but it is more common in young people. It can occur in any organ, most commonly involving soft tissue and the musculoskeletal system, such as the bone, periosteum, lymph nodes, and skin, but MS in the breast, ovary, rectum, pancreas, and bladder has also been reported in previous studies (1–5). MS involving the central nervous system is very rare, accounting for only 3.25% of MS cases (6, 7). The relationship between MS and hematopoietic diseases can be divided into four types (8):a) MS occurs simultaneously with AML; b) it occurs in chronic myeloproliferative disease; c) its first manifestation after remission of leukemia treatment; and d) it occurs without any leukemia patients whose manifestations are called primary or solitary MS.

Very few MS cases are associated with hematological diseases and affect only the central nervous system. They have only been mentioned in individual reports (11, 12).

The diagnosis of myeloid sarcoma (MS) is based on a comprehensive assessment of the patient’s clinical features, imaging characteristics, tissue biopsy, and immunohistochemistry. Compared to skeletal muscle signals, lesions have equal and lower signals on T1WI and higher signals on T2WI.The enhanced scan shows mild–to–prominent enhancement; however, some cases are not obvious. Therefore, the performance of MS on MRI is not specific (15).

Morphologically, MS lesion-infiltrating cells are classified as granulocytes or monocytes. Under a microscope, tumor cells are mostly diffusely distributed in a sheet shape, and the shape is more consistent and single, small to medium in size, consistent in size, with less cytoplasm, and lightly stained. Additionally, part of the cytoplasm is eosinophilic granular, nuclear round or irregular, with atypia, a thick nuclear membrane, visible nucleoli, a partially visible kidney–shaped nucleus, prominent mitotic figures, and scattered naive eosinophils in tumor cells (16, 17).

The diagnosis of MS depends on pathological examination. Lysozyme, MPO, CD43, andCD68 are the most common and widely expressed markers of MS. CD34 and CD117(c-Kit) are myeloid immature markers that can be partially positive in MS. Other markers include CD4, CD33, CD56, and terminal deoxyribonucleotide transferase (TdT) (18). Studies have indicated that. for both isolated MS and MS associated with AML, the recommended approach is systemic chemotherapy following the AML regimen, which can substantially extend leukemia–free remission and overall survival, Some studies have shown that chemotherapy regimens containing cytarabine are an important part of systemic chemotherapy for MS patients (17). Some studies have reported successful intrathecal chemotherapy combined with systemic chemotherapy for MS restricted to the nervous system.

The diagnosis of myeloid sarcoma (MS) poses a significant challenge to clinicians due to its rarity, particularly in cases where the tumor involves the central nervous system exclusively without evidence of systemic hematologic involvement. The patient was a 39-year-old male whose age was consistent with the age of onset of the disease. However, the clinical symptoms of this patient were very atypical at the time of onset, and the disease was misdiagnosed, When multiple abnormally enhanced signals were detected via MR and a large number of abnormal cells were detected via CSF cytology, due to a low level of awareness of the disease, histopathological examination did not involve examination of disease-specific markers, and the comprehensive results suggested that the intracranial lesions were inflammatory lesions, which caused the patient to lose the best opportunity for treatment. This finding suggests that although histopathological examination is still the gold standard for diagnosing tumors, cytological morphology is still highly suggestive of the diagnosis of the disease. Early CSF cytological analysis can detect abnormalities, and cell morphology and classification play significant roles in the differentiation of disease. In this case, cytological abnormalities were detected when the patient’s symptoms were mild, but they failed to attract sufficient attention, resulting in a missed opportunity for further diagnosis. CSF cytology is often used as an observation index for treatment efficacy in clinical practice. After intrathecal injection of antitumor drugs, the atypical cells in this patient decreased significantly. However, due to insufficient experience, greater attention should be paid to the significance of abnormalities detected in basic examinations to avoid missed or incorrect diagnoses and treatment delays. At the time of admission, the patient was critically ill, unconscious, and had markedly elevated intracranial pressure, with a constant risk of cerebral herniation. A lumbar cistern drainage was immediately performed to relieve the high intracranial pressure. Lumbar cistern drainage is an effective method for rapidly and safely reducing intracranial pressure and saving the lives of patients, particularly critically ill patients with encephalitis, whose intracranial pressure is often greater than 400 mmH, O. This procedure also provides valuable time for more accurate diagnosis at a later stage and offers new insights into the treatment of MS. In this patient ‘ s treatment, because there was no evidence of intracranial metastasis caused by extracranial malignancy, and the disease was considered a malignant tumor confined to the central nervous system, intrathecal injection of methotrexate was attempted for local chemotherapy. After eight courses of intrathecal chemotherapy, the patient ‘s clinical symptoms and CSF indices improved, and some cervical spinal cord lesions were slightly smaller, alleviating pain and prolonging survival. This case also provides useful experience for treating MS. As mentioned in previous reports, patients may benefit more from intrathecal chemotherapy combined with systemic chemotherapy. Although this patient later received systemic chemotherapy, the optimal timing was missed, leading to a poor prognosis. Through this case, a better understanding of MS was gained, along with valuable clinical experience. We hope that clinicians can learn from our work and pay closer attention to the interpretation of basic clinical examinations to avoid missed diagnoses and misdiagnoses. Timely diagnosis and appropriate treatment are essential for achieving favorable outcomes.

## Data Availability

The original contributions presented in the study are included in the article/Supplementary Material. Further inquiries can be directed to the corresponding author.
